# Computer-aided characterization of early cancer in Barrett’s esophagus on i-scan magnification imaging: a multicenter international study

**DOI:** 10.1016/j.gie.2022.11.020

**Published:** 2023-04

**Authors:** Mohamed Hussein, David Lines, Juana González-Bueno Puyal, Rawen Kader, Nicola Bowman, Vinay Sehgal, Daniel Toth, Omer F. Ahmad, Martin Everson, Jose Miguel Esteban, Raf Bisschops, Matthew Banks, Michael Haefner, Peter Mountney, Danail Stoyanov, Laurence B. Lovat, Rehan Haidry

**Affiliations:** 1 Division of Surgery and Interventional Sciences, University College London, UK; 2 Wellcome/EPSRC Centre for Interventional and Surgical Sciences (WEISS), University College London, UK; 3 Department of Gastroenterology, University College London Hospital, UK; 4 Odin Vision, UK; 5 Department of Gastroenterology and Hepatology, Clínico San Carlos, Madrid, Spain; 6 Department of Gastroenterology and Hepatology, University Hospitals Leuven, Leuven, Belgium; 7 Krankenhaus der Barmherzigen Schwestern, Department of Internal Medicine II, Vienna, Austria

## Abstract

**Background and aims:**

We aimed to develop a computer-aided characterization system that could support the diagnosis of dysplasia in Barrett’s esophagus (BE) on magnification endoscopy.

**Methods:**

Videos were collected in high-definition magnification white-light and virtual chromoendoscopy with i-scan (Pentax Hoya, Japan) imaging in patients with dysplastic and nondysplastic BE (NDBE) from 4 centers. We trained a neural network with a Resnet101 architecture to classify frames as dysplastic or nondysplastic. The network was tested on 3 different scenarios: high-quality still images, all available video frames, and a selected sequence within each video.

**Results:**

Fifty-seven patients, each with videos of magnification areas of BE (34 dysplasia, 23 NDBE), were included. Performance was evaluated by a leave-1-patient-out cross-validation method. In all, 60,174 (39,347 dysplasia, 20,827 NDBE) magnification video frames were used to train the network. The testing set included 49,726 i-scan-3/optical enhancement magnification frames. On 350 high-quality still images, the network achieved a sensitivity of 94%, specificity of 86%, and area under the receiver operator curve (AUROC) of 96%. On all 49,726 available video frames, the network achieved a sensitivity of 92%, specificity of 82%, and AUROC of 95%. On a selected sequence of frames per case (total of 11,471 frames), we used an exponentially weighted moving average of classifications on consecutive frames to characterize dysplasia. The network achieved a sensitivity of 92%, specificity of 84%, and AUROC of 96%. The mean assessment speed per frame was 0.0135 seconds (SD ± 0.006).

**Conclusion:**

Our network can characterize BE dysplasia with high accuracy and speed on high-quality magnification images and sequence of video frames, moving it toward real-time automated diagnosis.

## Graphical abstract

**Figure:**
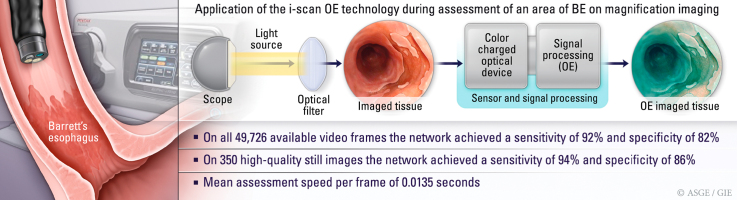

There is a global rise in the incidence of esophageal adenocarcinoma (EAC) affecting 0.7 per 100,000 person-years, predominantly in Western countries.^1^ Barrett’s esophagus (BE) is associated with an increased risk of progression from nondysplastic BE (NDBE) to low-grade dysplasia (LGD) to high-grade dysplasia (HGD) to EAC.^2^

Despite advances in endoscopic technology, EAC in BE is still underdiagnosed.^3^ A multicenter study showed an overall missed esophageal cancer rate of 6.4%.^4^ One factor associated with a missed diagnosis is endoscopist’s experience. Computer-aided diagnosis (CAD) and characterization can potentially help offset some of these factors.

The endoscopic evaluation of BE dysplasia is a 2-step process whereby the BE segment is initially assessed in overview in high-definition white-light imaging modes. A more detailed inspection is then performed with chromoendoscopy and magnification imaging when a lesion is identified, which provides improved visualization of the mucosal architecture and vasculature.^5^ This is particularly useful in assessing resection margins, allowing for a complete resection (R0). Assessment on magnification imaging may be difficult for nonexpert endoscopists even though classification systems are available.^6^, ^7^, ^8^, ^9^, ^10^, ^11^, ^12^

The i-scan optical enhancement (OE) system (Pentax, Hoya, Japan) uses preprocessing and postprocessing techniques to allow surface enhancement of the superficial mucosal vascular structures. Using an optical filter, OE delivers specific wavelengths of light that correspond to the main absorption spectrum of human hemoglobin, which allows enhancement of the microvasculature within the most superficial layers of the mucosa (Fig. 1). The use of magnification endoscopy combined with OE allows closer inspection (×≤136 resolution) of the mucosal structures and vasculature.^13^

**Figure 1:**
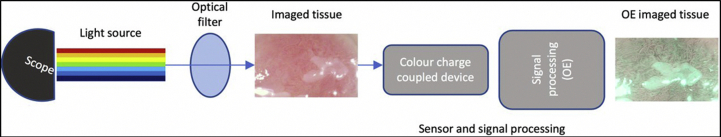
Before and after application of the i-scan optical enhancement (OE) technology during assessment of an area of Barrett’s esophagus on magnification imaging. Image courtesy of Everson et al, 2019.^13^

Lipman et al^12^ validated the use of previous i-scan systems for the characterization of dysplasia in BE using a simple classification system based on mucosal and vascular patterns. Using this classification system, Everson et al^13^ showed that the accuracy of neoplasia detection was significantly higher when experts used OE versus high-definition white-light (HD-WL) magnification (84% vs 77%).

Struyvenberg et al^5^ developed a CAD system with promising diagnostic accuracy in predicting the presence of dysplasia in BE on narrow-band imaging (NBI) zoom videos using the Olympus system. As far as we are aware, that was the only published study to develop a neural network for characterization on magnification imaging in BE. To our knowledge, no studies have developed a CAD system for magnification imaging on the Pentax OE/i-scan 3 imaging system. A computer-aided detection system will help detect areas of dysplasia in BE in overview assessments of the esophagus. A CAD system would supplement a computer-aided detection system to further confirm and classify the pathologic features of an area of BE and confirm clear resection margins.

The primary aim of this international multicenter study was to develop a novel CAD system that could characterize and diagnose BE dysplasia on OE/i-scan3 magnification endoscopic imaging on 3 levels by assessing the sensitivity, specificity, and accuracy of the model on (1) high-quality still images, (2) short sequence of frames, and (3) real time on whole videos.

The secondary aims were to assess the speed of the networks in characterizing dysplasia.

## Methods

### Patient recruitment

Patients attending for BE assessment at 4 expert European centers were recruited. All cases were collected prospectively, including cases collected prospectively as part of a previous BE imaging study.^13^ Patients with esophageal strictures, varices, and ulceration were excluded from recruitment. The study was approved by the Cambridge central research ethics committee (REC Reference No. 18/EE/0148) for UK sites. European centers received ethical approval from local committees for the use of images for this and other imaging-based research projects.

### Endoscopic procedures and video collection

Videos were collected by 4 expert endoscopists (R.J.H., R.B., J.M., M.H.). All those endoscopists had >10 years’ experience in the assessment of BE, performed BE endotherapy weekly, and had access to zoom endoscopes in their units to perform magnification endoscopy.

Magnification videos were prospectively collected by use of the Pentax endoscopy system. Mucus lining the esophagus was removed with a simethicone and water solution. Endoscopists performed a pull-through, which involved slow withdrawal of the endoscope from the gastroesophageal junction to the maximal extent of BE in high-definition white-light (HD-WL) imaging. If a lesion was identified, it was assessed with a ×136 zoom by use of HD-WL, i-scan1, and i-scan3/OE. If there was no lesion or dysplasia, the endoscopist selected 1 normal area and assessed it with zoom imaging, which was recorded.

### Tissue acquisition and histologic analysis

Areas suggestive of dysplasia and assessed with magnification imaging underwent a target biopsy or EMR. The EMR samples were affixed to a cork board with pins and needles and then embedded in paraffin in the histopathology laboratory. Areas of BE with no suspicion of dysplasia on magnification imaging underwent target biopsy. The results of histologic examination showing dysplasia were reviewed by 2 histopathologists with expertise in BE and >10 years of experience in each center.

### Annotation strategy

A computer vision annotation tool (Odin vision, London, UK) was used to annotate a sequence of magnification video frames as dysplasia/no dysplasia. The area of annotation was matched with the resection or biopsy areas. Annotations were done on a frame level for the presence or absence of dysplasia based on the mucosal pit pattern and vasculature. The criterion standard was determined by the results of the histological analysis of the biopsy specimen or EMR specimen. The frames were used to train and validate a convolutional neural network (CNN) to characterize dysplasia on magnification imaging.

### Model data set

In all, 57 patients were included (23 NDBE patients, 34 dysplastic patients). To train the network, 60,174 (39,347 dysplasia, 20,827 NDBE) magnification video frames were used. These images included white light, i-scan1, and i-scan3/OE images. Performance was evaluated by a leave-1-patient-out crossvalidation methodology with a test set that included all the i-scan3/OE frames only, reflecting a real-world scenario whereby assessment on zoom is done with chromoendoscopic imaging. This means that 57 different models or folds were generated. Each model was trained with all procedures except 1, which became the test case. Therefore, the AI system was tested on all the cases. The testing set included all 49,726 i-scan3/OE magnification frames (35,262 dysplastic frames, 14,464 nondysplastic frames). Table 1 shows a breakdown of the data set based on site location and histologic features of the lesions.

**Table 1:** Breakdown of the data set based on location and histopathologic features

	Location				Histopathologic feature		
	United Kingdom	Belgium	Spain	Austria	HGD	IMC	NDBE
Number of patients	30	17	6	4	19	15	23

*HGD*, High-grade dysplasia; *IMC*, intramucosal adenocarcinoma; *NDBE*, nondysplastic Barrett’s esophagus.

Three levels of test data sets were created to allow 3 different levels of results, which can be interpreted differently depending on the tailored requirements of the artificial intelligence (AI) system: (1) high-quality still images, (2) short continuous sequence of frames within a video, and (3) all available video frames.

#### Test set 1: high-quality still images

**Figure 2:**
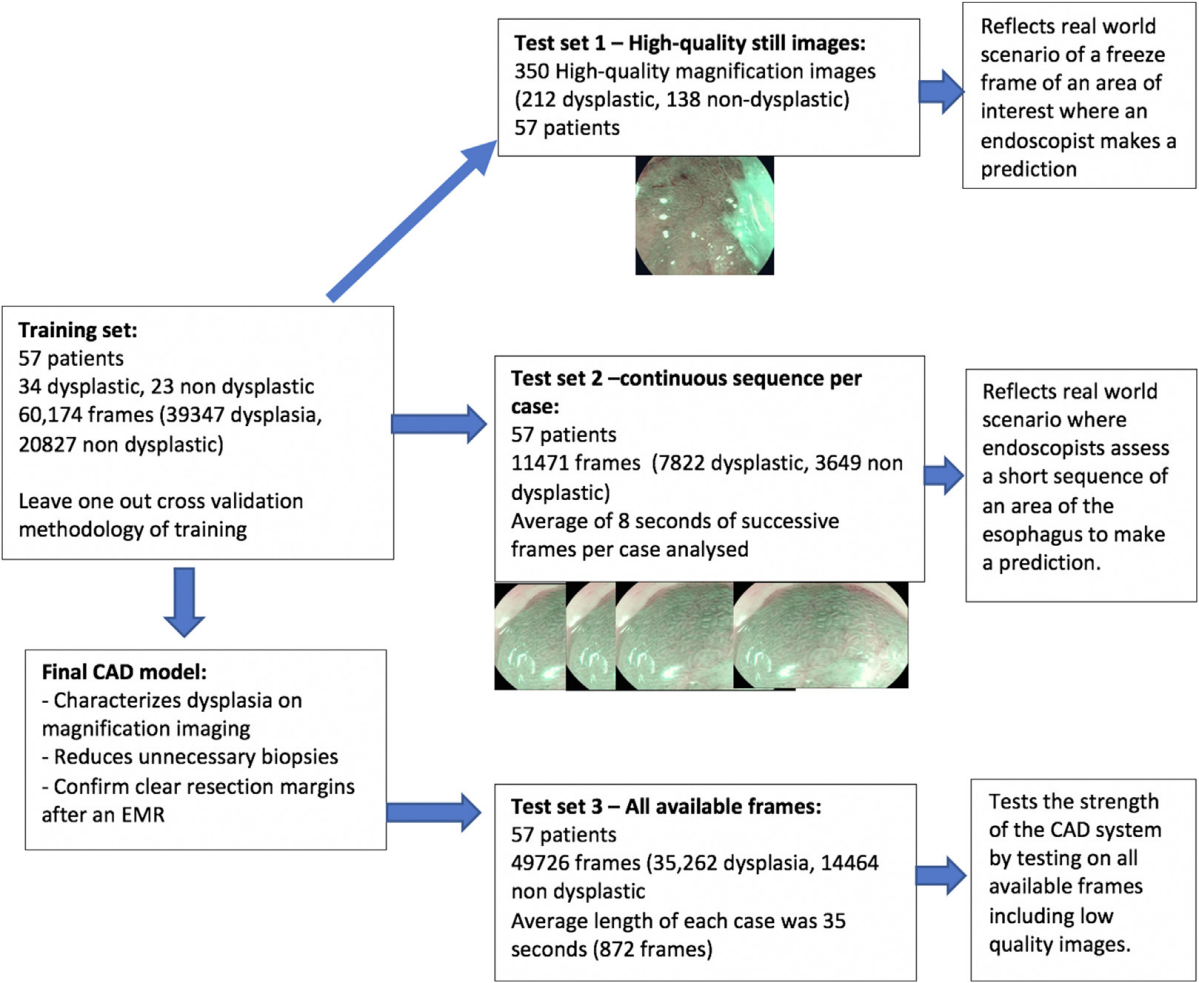
Breakdown of the data set and potential importance of each level of testing data set in the computer-aided diagnosis (CAD) system output.

Between 5 and 16 high-quality still OE/i-scan3 images were randomly selected from each patient. There was a total of 350 images (212 dysplastic, 138 nondysplastic) in this test set. This reflects a possible scenario whereby the endoscopist would first capture a high-quality magnification image on a freeze frame on which an assessment is then made (Fig. 2).

#### Test set 2: sequence of frames

A short continuous sequence of frames was annotated for the presence or absence of dysplasia. A random part of each video was selected. There was a total of 11,471 i-scan3/OE frames in this test set. The average length of each sequence was 8 seconds per patient. In reality, experts would spend a short time assessing an area of interest on magnification imaging. This provides an experiment that reflects the real-world scenario in how an assessment is made on zoom imaging. In the same way experts make better decisions by inspecting the lesion in several frames, this test set allows us to evaluate the AI system while allowing for a temporally informed decision (Fig. 2).

#### Test set 3: all video frames

All available 49,726 i-scan3/OE frames were included. No frames were excluded, even lower-quality images. The average length of each case recording was 35 seconds (872 frames) per patient (Fig. 2).

### Classification convolutional neural network

#### Preprocessing and augmentation

A CNN was trained with a ResNet101 architecture to characterize BE video frames as dysplastic or nondysplastic by use of a leave-1-patient-out cross-validation methodology. We trained 57 models on all the procedures except 1, which becomes the test procedure for that fold. Each fold was tested on the same epoch to ensure generalization across the data.

Data augmentation was performed randomly to reduce overfitting, including color transformations (brightness, contrast, saturation, and hue) and affine transformations (rotation, translation, and scaling). A validation set was used after each training iteration on 5000 images to spot for divergence in the validation loss. The training parameters were kept the same for each fold. Minibatch training was done whereby a training iteration is performed with different minibatches of 5000 images. Images were cropped (removing the black borders) and then resized to 448 × 448 pixels, and then the pixel values were normalized. Data leakage was prevented by ensuring that all images from each patient were always in the same set (training or testing) on each iteration.

#### Hyperparameters and training

The network was pretrained on ImageNet and then fine-tuned on our data. Learning rate: 1e-4, fine-tuned for 6 epochs, minibatch size 32. The pretrained model was trained with Pytorch, the same as our model. The ImageNet weights are provided in the Pytorch platform.

#### Postprocessing

The model was trained to classify over 2 classes: dysplastic and nondysplastic. The output of the model was the probability of dysplasia for each frame, a number between 0 and 1 that was then thresholded. On the prediction on the sequence of frames (test set 2), an exponentially weighted average of the consecutive frames was used to make a diagnosis of dysplasia. The same threshold of .65 was used on each testing set. The processing speed of the model was measured on an NVIDIA GeForce RTX 3090 graphics processing unit.

### Statistical analysis

Descriptive statistics consisted of the mean (± standard deviation). The performance of the CAD system on a per-frame and per-patient level was calculated in terms of accuracy, area under the curve (AUC), specificity, and sensitivity. A 57-fold leave-1-patient-out cross-validation methodology was used to train and assess the performance of the CNN.

## Results

### Test set 1: high-quality still images

**Figure 3:**
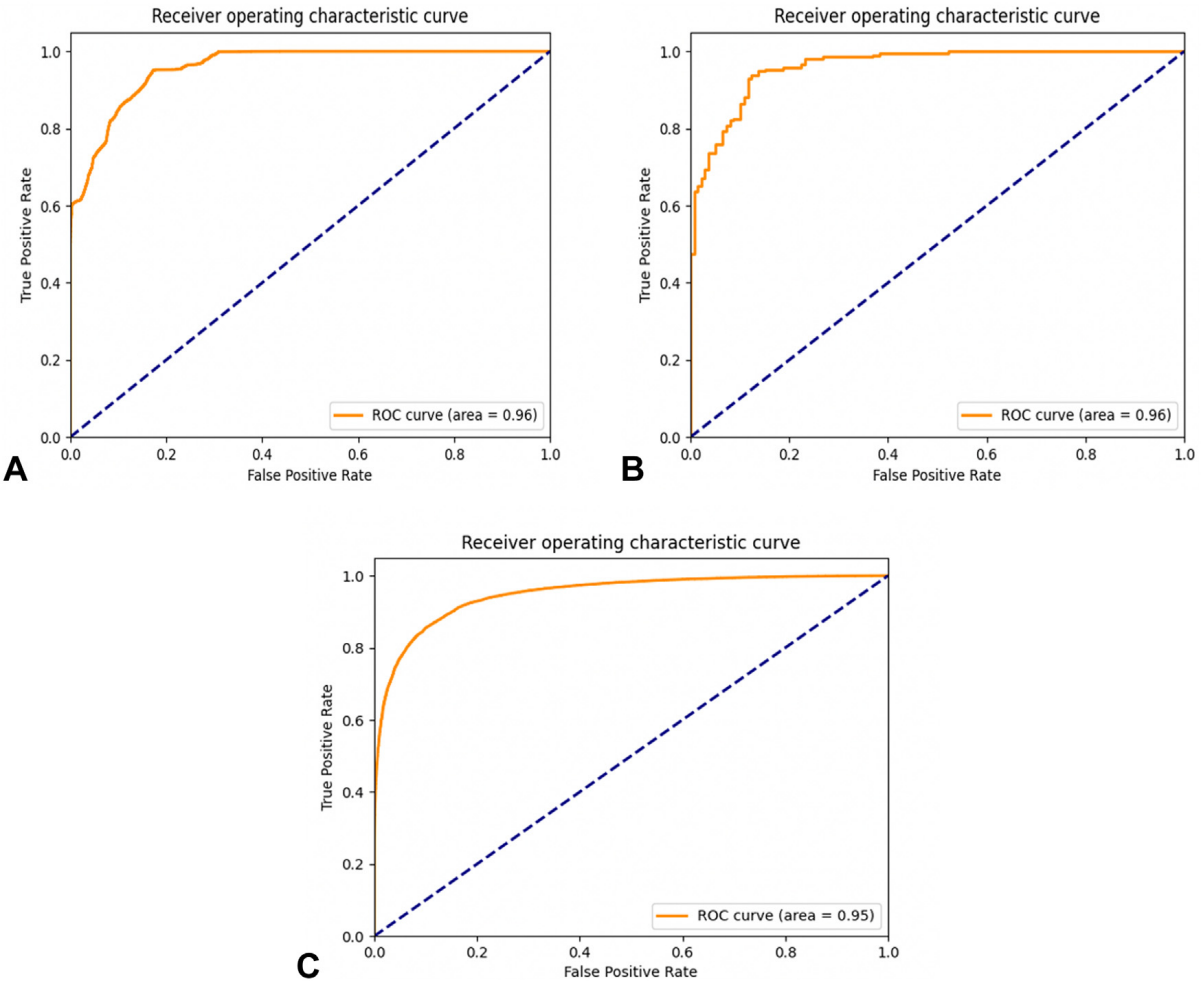
Area under the receiver operating characteristic (ROC) curve performance of the computer-aided diagnosis system on (**A**) 350 still images, (**B**) consecutive sequence of frames per case, and (**C**) 49,726 i-scan3/optical enhancement (all available) frames.

The CAD system was tested on 350 high-quality i-scan3/OE still images, resulting in a per frame accuracy of 91%, sensitivity of 94%, specificity of 86%, and AUC of 96% in characterizing dysplasia (Fig. 3).

### Test set 2: sequence of frames

The CAD system was tested on a total of 11,471 frames by using an exponentially weighted moving average, achieving an accuracy of 90%, sensitivity of 92%, specificity of 84%, and AUC of 96% (Fig. 3).

**Figure 4:**
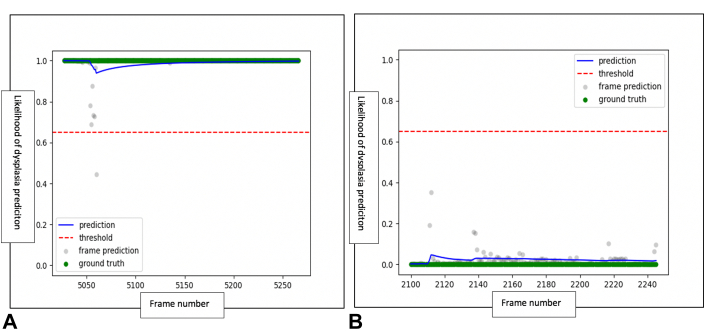
Using an exponentially weighted moving average, the computer-aided diagnosis (CAD) system predicts the likelihood of neoplasia on i-scan3/optical enhancement magnification imaging over a consecutive sequence of frames. **A,** The CAD system correctly predicts the likelihood of neoplasia (*blue line*) on each frame. This overlaps with the prediction of the endoscopist (*green line*) and the criterion standard (histologic analysis). **B,** The CAD system correctly predicts a 0% likelihood of neoplasia in this case of nondyplastic Barrett’s esophagus.

Figure 4 shows an automated video analysis of a dysplastic and a nondysplastic case on magnification endoscopy. It shows the likelihood of a correct prediction of dysplasia/nondysplastic by the CAD system per frame and compares the performance with the endoscopists’ predictions. Temporal filtering was then used to make a per-case prediction.

### Test set 3: all video frames

The CAD system was tested on all 49,726 i-scan3/OE frames, achieving a per-frame accuracy of 89%, sensitivity of 92%, specificity of 82%, and AUC of 95% (Fig. 3).

### CNN performance per patient

#### Test set 2: sequence of frames

In a per-patient analysis based on the sequence of frames, the CAD system had a sensitivity of 91% and specificity of 78% in characterizing dysplasia (based on a threshold of >80% of frames in each video being predicted correctly).

Three different scenarios for per-patient prediction were generated based on different correct prediction thresholds. These can be adjusted in an endoscopic system. The optimal scenario would be number 3 (Table 2). In this situation, in 97% of the videos, >70% of the frames were correctly predicted as dysplasia.

**Table 2:** Different scenarios for a per-patient prediction based on the proportion of frames in a sequence that correctly predict dysplasia

Scenario	Proportion of frames in a sequence that correctly predict dysplasia	Per-patient sensitivity
1	>90%	85%
2	>80%	91%
3	>70%	97%

#### Test set 3: all video frames

On a per-patient analysis of all available frames, the CAD system achieved a sensitivity of 91% and specificity of 70% (based on a threshold of >70% of frames in each video being predicted correctly).

### CNN performance based on histologic features

#### Test set 2: sequence of frames

Out of the 34 cases of dysplastia, 19 were HGD and 15 were intramucosal adenocarcinoma (IMC) on histologic analysis. Based on the sequence of frames per case analysis, the CAD system had a sensitivity of 88% in characterizing HGD (3933 frames out of 4485 correctly predicted) and 99% in characterizing IMC (3287 frames out of 3337 correctly predicted). The CNN achieved a per-frame specificity of 84% in the 23 patients with no dysplasia on magnification imaging.

#### Test set 3: all available frames

The CAD system had a sensitivity of 92% in characterizing IMC (12,613 out of 13,722 frames correctly predicted as dysplastic) and a sensitivity of 92% in characterizing HGD (19,924 out of 21,540 frames correctly predicted as dysplastic). The CNN achieved a per-frame specificity of 82% on the nondysplastic cases.

### Speed of characterization of dysplasia in BE

**Figure 5:**
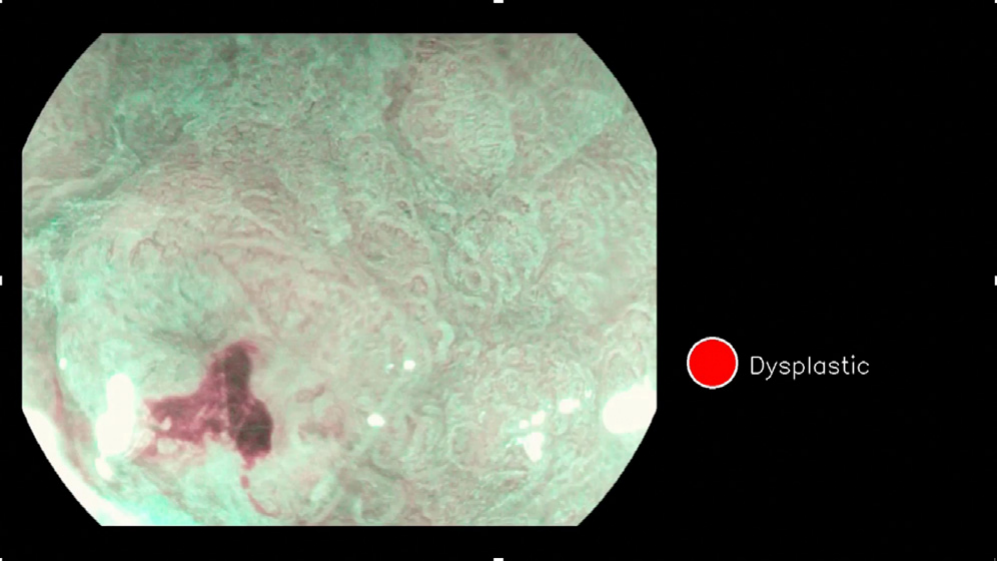
The artificial intelligence system correctly predicts an area of dysplasia on optical enhancement magnification.

The mean assessment speed per frame was 0.0135 seconds (SD ±0.006) or 74 frames per second. Figure 5 shows an example of the system being used to correctly predict a diagnosis of dysplasia on a particular frame in OE.

## Discussion

This study demonstrates a CAD system for the diagnosis and characterization of dysplasia on magnification chromoendoscopy imaging with high sensitivity and specificity, both on high-quality images and on a high number of successive frames. The results suggest that this algorithm may work effectively in real time in the live endoscopy setting.

To the best of our knowledge, this is the first study to demonstrate the performance of an AI algorithm on Pentax (i-scan3/OE) magnification imaging. The only other study to develop a CAD algorithm for diagnosis on magnification imaging was developed using NBI. That study reported promising diagnostic accuracy; on 30,021 NBI magnification video frames, the CAD demonstrated a sensitivity and specificity of 85% and 83%, respectively.^5^ On our study, on 49,726 OE magnification frames the AI system achieved a sensitivity and specificity of 92% and 82%, respectively, in characterizing dysplasia. It is difficult to make a direct comparison between the 2 networks because they were developed by the use of 2 different imaging systems. Further studies are required to develop a network that is able to generalize across all endoscopic platforms.

Several studies have developed classification systems for the characterization of BE, and they are all based on magnification endoscopy.^6^, ^7^, ^8^ Magnification imaging allows for a clearer distinction of the pit pattern and vascular abnormalities, which would allow a clear differentiation of dysplasia versus no dysplasia to be made. However, these classification systems have complex criteria, meaning that there is not a large uptake by nonexpert endoscopists.^14^ An AI system for characterization will help make this process much easier in nonexpert hands by aiding these predictions to be made on magnification imaging.

Our group recently published an article describing an AI system that is able to detect and localize BE dysplasia with targeted biopsies on overview images.^15^ Other groups have also published promising results for the detection of dysplasia in BE.^16^, ^17^, ^18^, ^19^ These detection systems would be more relevant to the general endoscopist in improving detection rates because they do not require specialist equipment or classification criteria. By contrast, these magnification AI systems would be very useful to experts, in particular helping to delineate lesion margins and achieve an R0 resection and therefore minimizing the risks of recurrence. The 2 features of an AI system with detection and characterization can work together as part of a 2-stage algorithm to help achieve optimal outcomes for patients.

We developed 3 different iterations of test sets to show the ability of the CAD system to tailor to different scenarios and to test its performance across different conditions to diagnose dysplasia. We tested performance on high-quality still images (n = 350 images), sequence of frames within each case, and all the available magnification frames in the videos, including lower-quality images. One can argue that the most clinically relevant factor is the performance on high-quality still images. When an endoscopist performs an assessment on magnification imaging, areas of interest are captured with a freeze frame and visually assessed in this modality. The AI system can seamlessly make a prediction at the same time as an endoscopist’s assessment on this freeze frame image. The continuous sequence of frames within each case reflects the real-world scenario whereby an endoscopist assesses a specific part of the esophagus on magnification imaging. In the same way experts make better decisions by inspecting the lesion in several frames, this test set allows us to evaluate the AI system while allowing for a temporally informed decision. The AI system had a high performance on these frame sequences (92% sensitivity, 84% specificity). Prospective randomized control trials are required to test these findings.

The system was trained on multiple video frames to maximize the ability of the CNN to work in different environments. This is why we developed 3 iterations of testing data sets to allow us to test the performance of the model in different scenarios. A leave-1-out cross-validation methodology was used. Owing to the subtlety of the lesions and the limited data set, models trained with different train/test/validate splits generate results that vary greatly and could imply statistical uncertainty about the estimated performance. If an experiment yields low results, it is hard to know whether the model did not learn a good-enough representation. Therefore, we decided to use leave-1-out cross-validation, so all the patients can be evaluated separately by use of a model as close as possible to training with the whole data set. This methodology allows seeing the potential of the model with limited data.^20^

The system was able to diagnose dysplasia on magnification imaging with a speed of 0.0135 seconds per frame. In the only other similar study, the processing speed of the AI system was 0.026 seconds per frame.^5^ To fairly compare the speed of different systems, the systems would need to be benchmarked on the same machine. The results show that the system would be able to work in real time to support the decision making of endoscopists on assessments on magnification imaging.

A particular strength of the study was in the inclusion of several different centers and the various histologic features. The CAD system was able to perform well on HGD, intramucosal adenocarcinoma, and NDBE. These strengths potentially will provide more favorable results with the system if tested in different countries and settings. This is something that can be tested with a prospective trial.

**Figure 6:**
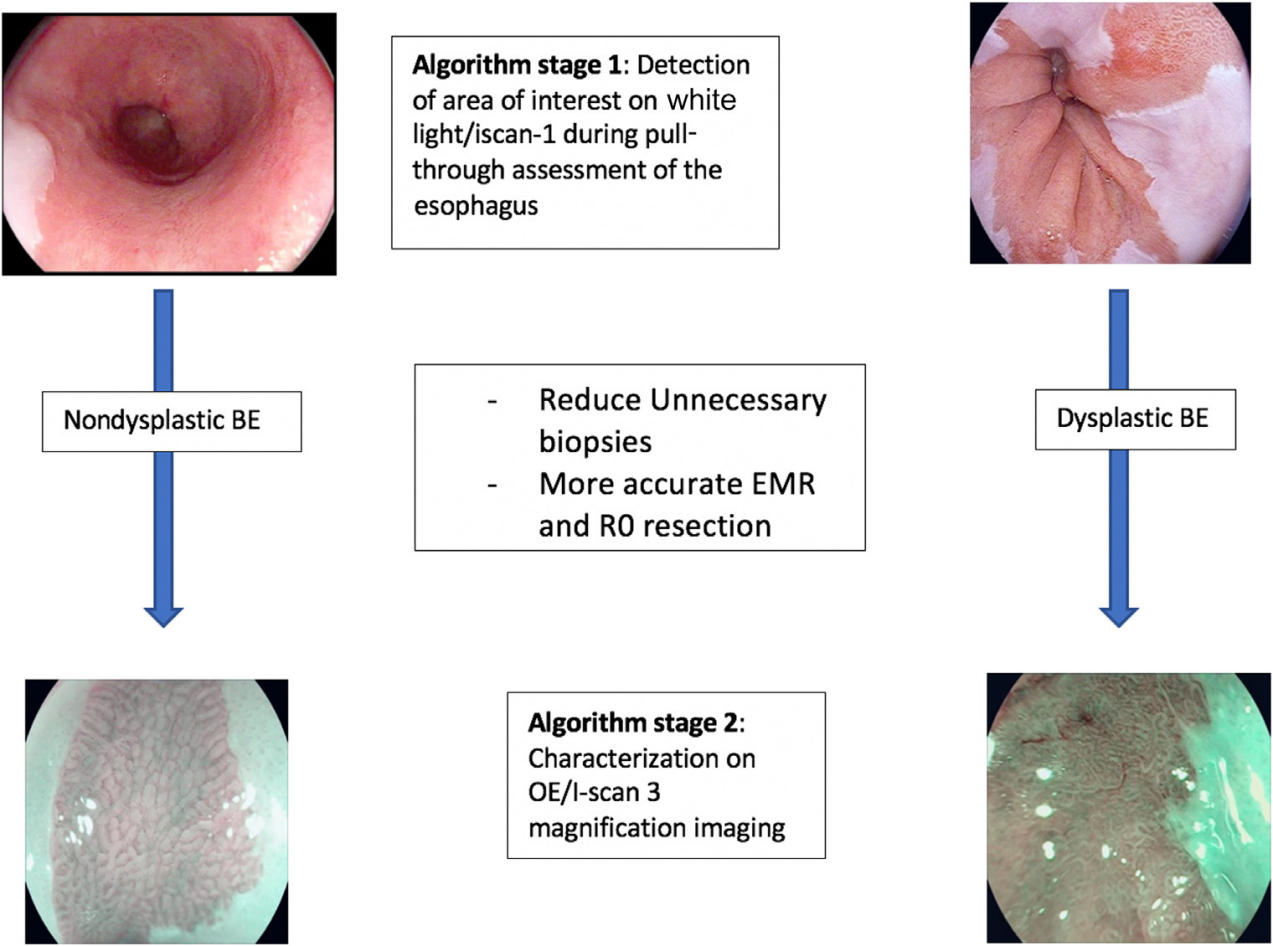
A 2-stage algorithm for detection of dysplasia on overview pull-through assessment of the esophagus on white-light/i-scan1 imaging followed by characterization on i-scan3/optical enhancement (OE) magnification imaging. *BE*, Barrett’s esophagus.

This CAD system would fit in as part of a 2-stage algorithm. The first step would be detection of suggestive areas during a pull-though assessment of the esophagus on white light/i-scan1 images. Our group recently published a study of this problem, whereby the detection algorithm would identify an area of abnormality for a targeted biopsy.^15^ This would be relevant to all hospitals that perform assessment of BE. The second step of the algorithm would be the step discussed in this study to characterize and diagnose the area of abnormality once identified. This would help confirm further an area of dysplasia and would also help with delineation of the resection margins to ensure an R0 resection of the lesion, offering curative endoscopic therapy to patients. This second step would be particularly relevant to experts in tertiary referral centers and also to district general hospitals that have zoom endoscopes in helping to confirm an area of dysplasia (Fig. 6).

There are limitations to this study. We developed a CAD system using videos from a single endoscopic system. In future studies we plan to develop a system based on imaging from multiple platforms so it can be generalized across all systems. Another limitation is that the AI system was not benchmarked against endoscopists. We plan to do this in future studies and to test the performance of the system against expert and nonexpert endoscopists using an external data set. We believe that this model will have different roles depending on the expertise of endoscopists with the use of zoom imaging in the assessment of BE. For nonexperts it will be helpful in confirming the diagnosis of dysplasia, whereas for experts it will help with delineating the resection margins for EMR, helping ensure an R0 resection. Another limitation is that we included lower-quality images in the training set. The CAD might have been more robust if we had included only a high-quality sequence of frames, given that realistically, on magnification imaging, endoscopists make their assessments on these high-quality images. We included the lower-quality images to potentially maximize the ability of the model to work in the real-world endoscopy setting. A limitation of this study is that there was no final test from an external data set of images. This would help strengthen our results, and we plan to do this in future studies. This could be potentially done as part of a clinical trial that would test the real-time applicability of this model. In terms of histologic features, no LGD was included in this phase of the study. This is something we plan to include in future studies because these cases are more difficult to characterize and would provide further value to this model. We would require training a model with a large volume of LGD dysplasia cases to be able to then characterize them.

We present a CAD system that is able to diagnose and characterize dysplasia on magnification imaging with high accuracy on both a per-frame and a per-patient level. This would allow the potential reduction of unnecessary biopsies and more accurate EMR results, minimizing the risk of recurrence. The system would need to be validated in a prospective multicenter randomized control trial in a real-time endoscopic setting.
